# Sinus Balloon Dilation as Treatment for Acute Sphenoid Sinusitis with Impaired Vision for a Child

**DOI:** 10.1155/2016/5209243

**Published:** 2016-02-24

**Authors:** Yin Zhao, Kangbing Chen, Zonggui Wang

**Affiliations:** Department of Otolaryngology-Head and Neck Surgery, The Second Hospital of Jilin University, Changchun 130041, China

## Abstract

This paper is about sinus balloon dilatation in treatment of acute left sphenoid sinusitis with left impaired vision in a child. Balloon catheter dilatation (BCD) of the sinus ostia is a new technique. It has been shown to be a minimally invasive technique to manage chronic sinusitis. However, this method is rarely used in the treatment of acute sinusitis. So far, we know of no reported cases of sinus balloon dilatation in treatment of this case, especially for children.

## 1. Introduction

Acute sphenoid sinusitis can result in symptoms such as headache, characteristically creating deep or vertex pain, nausea, and even orbital complications. Orbital complication of acute rhinosinusitis typically affects children and young adults [1]. Visual disturbances are rare but serious. Anatomically, there is only a thin plate of bone that separates the optic nerve from the sphenoid sinuses, and hence sphenoid sinus inflammation can infringe the optic nerve and cause retrobulbar optic neuritis. Major clinical manifestations include decreased vision or loss. Furthermore, delayed diagnosis in all age groups is a threat to both vision and life [2]. Therefore, acute sphenoid sinusitis with impaired vision is regarded as a medical emergency. Following adequate medical management, surgical ostial opening and drainage are usually indicated with the use of endoscopic sinus surgery (ESS). But standard endoscopic sinus surgery may need to alter the nasal and sinus anatomy for access. This might happen especially in children whose anatomy is small and limited. There is also some concern about impact of endoscopic surgery on facial growth in childhood [3]. In addition, serious complications such as haemorrhage, CSF leak, or orbital damage are reported for ESS [4]. More recently, balloon dilation of sinus ostia has emerged as a new tool in the armamentarium for endoscopic sinus surgery. Sinus balloon dilation may offer a more safe, expedient, and effective treatment method compared with traditional endoscopic sinus surgery for chronic rhinosinusitis [5]. However, to our knowledge, there have been no published reports on the feasibility of balloon dilation in the treatment of acute sphenoid sinusitis, especially in children. We describe the use of the new minimally invasive technique called balloon sinuplasty in the treatment of an acute sphenoid sinusitis with impaired vision in a child.

## 2. Case Report

**Figure 1:**
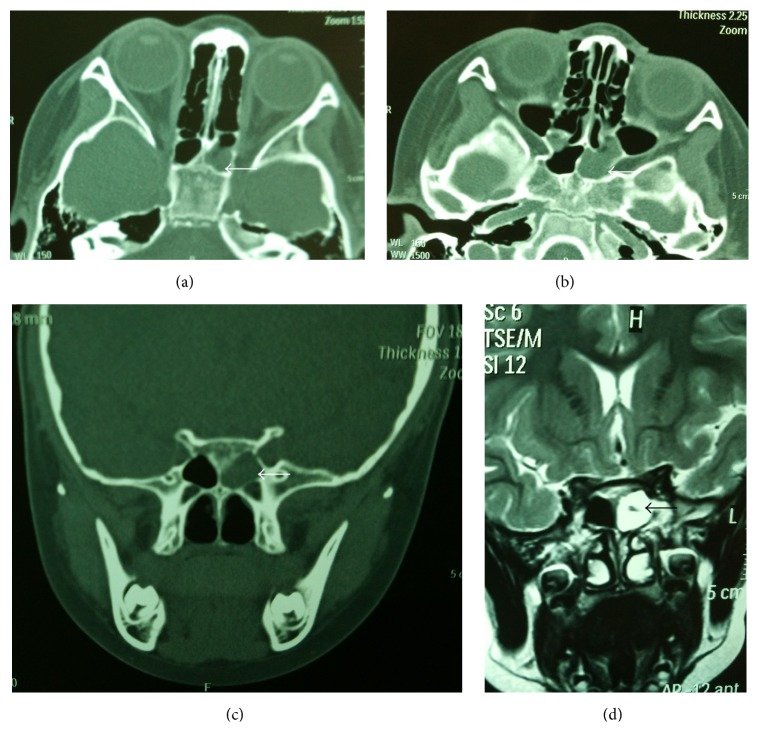
(a and b) Axial CT scans showing only sphenoid sinus with soft tissue density shadows on the left (*white arrow*). (c) Coronal CT scans of a patient with soft tissue density shadows in the left sphenoid sinus (*white arrow*). (d) T2-weighted MRI shows hyperintensity in the left sphenoid sinus (*black arrow*).

A previously healthy 5-year-old girl experienced sudden decreased left visual acuity lasting for seven days followed by left eye swelling. She was presented for initial evaluation in the Ophthalmology Department. Oculoplastic examination revealed no left proptosis, left eye 6/12 visual acuity and right eye 6/6 visual acuity, and normal perimetry and colour vision. Computed tomography (CT) and magnetic resonance imaging (MRI) scan of the orbits and paranasal sinuses showed only the left sphenoid sinus with soft tissue density shadows, but with no clear bone destruction (Figure 1). Because of the altered visual acuity and isolated sphenoid sinusitis, the ophthalmologist advised the patient to be evaluated by the Otolaryngology Department. Of interest, the patient lacked the typical symptoms of acute sphenoid sinusitis. She denied headache; diplopia; or history of sinusitis, nasal congestion, or purulent nasal discharge. Rhinoscopy revealed no mucopus secretions and nasal polyps in the nasal cavity. Following ophthalmologic and otolaryngologic evaluation and considering the patient's age and decreased vision, the patient was urgently managed with surgical dilation ostium of the left sphenoid sinus using endoscopic balloon sinuplasty (Acclarent, Inc., Menlo Park, CA).

### 2.1. Surgery

**Figure 2:**
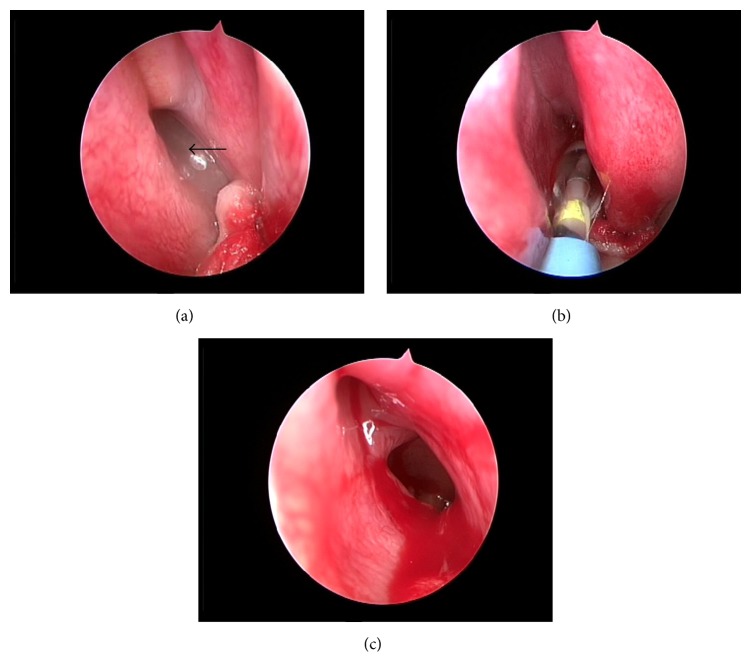
(a) Endoscopic visualisation of mucosal edema in the left sphenoethmoidal recess during operation (*black arrow*). (b) Balloon dilating sphenoid sinus ostium. (c) Larger sphenoid sinus ostium after dilating and mucosal edema in the sinus.

The patient was placed under general anaesthesia. Under endoscopic visualisation, mucosal edema was observed in the left sphenoethmoidal recess and sphenoid sinus ostium. A probe was used to confirm the sphenoid sinus ostium. A 30° guide catheter was passed along the path into the sphenoid sinus and a guide wire advanced into the sphenoid sinus. Once in the correct position, balloon was advanced over the guide wire and its position within the ostia was verified using endoscopic visualisation. The balloon was subsequently inflated to 12 atmospheres for ten seconds and sphenoid sinus ostium was visualised dilating. Severe mucosal edema was observed within the left sphenoid sinus. An irrigation catheter was used to flush the sinus with 2 mL pulmicort respules and 20 mL normal saline (Figure 2). There is no hemorrhage intraoperatively and filling postoperatively. After procedure, good ventilation of the ostia was visualised.

### 2.2. Follow-Up

The patient had an uneventful postoperative period and remained pain-free with no residual swelling of the nasal cavity. The patient's vision was improved in the morning of first postoperative day. Oculoplastic examination revealed her left eye 6/6 visual acuity and right eye 6/6 visual acuity and normal perimetry and colour vision. She was discharged home 24 hours postoperatively with 40 mg of methylprednisolone for 3 days, mometasone spray for 2 weeks. At a 2-month follow-up, the patient remained asymptomatic, well, and with normal vision.

## 3. Discussion

Acute sinusitis is predominantly an infectious disease caused by bacteria such as* Streptococcus pneumoniae*,* Haemophilus influenzae*, and* Moraxella catarrhalis*. The combination of both host and disease factors may result in the spread of infection beyond the paranasal sinuses. The orbit is particularly prone, and this is more so in children. Because there is only a thin plate of bone that separates the optic nerve from the sphenoid sinuses, the thickness about 0.5 mm, infection could spread to the orbit from either direct extension or a defect in the thin wall [6]. Orbital complication is regarded as a medical emergency. In our case, the patient was only a 5-year-old girl, who had suffered from asymptomatic acute left sphenoid sinusitis with left impaired vision for seven days. Her condition had not got any improvement during her illness. Because of the lack of improvement, we immediately opted for surgery to treat her. In the past, endoscopic sinus surgery (ESS) was used to open blocked ostia of sphenoid or ethmoid sinus to manage this condition. Unfortunately, problems with ESS still exist especially in those where too much tissue removal has been performed to damage normal anatomy of sinus and sinus drainage mucosa. Moreover, the locus of sphenoid sinus is deep and secluding. Serious complications of ESS for sphenoid sinus may occur, such as haemorrhage and CSF leak, even other orbital and intracranial complications [4]. Consequently, we should contemplate cautiously taking this procedure in children.

Balloon catheter dilation is a technique which has revolutionized sinus surgery in recent times. The benefits of using the balloon catheter dilation technology include preservation of the normal anatomy of the vital ostiomeatal complex, while precisely focusing on the occluded sinus ostium and the diseased sinus cavity beyond it [7]. It allows the operator to widen the natural ostia to allow for natural drainage rather than resecting the normal anatomy to achieve the result, thus avoiding serious complications. Then, it is relatively mucosal sparing. Hence, blood loss can often be minimized. Recent literature has documented clear indications for the sinus balloon dilation surgery. Ideal candidates for this procedure would include chronic sinusitis limited mostly to ostial obstruction of the frontal, maxillary, and sphenoid sinuses, with near normal middle meatal integrity. A recent prospective study of 195 sinus ostia dilated with balloons showed that patients who underwent balloon dilation during endoscopic sinus surgery showed significant symptom improvement as well as radiographic confirmation of disease resolution 2 years postoperatively, showing the durability of the hybrid procedure [8]. In the study of 32 children with CRS by Ramadan et al., they showed that balloon catheter dilation of the sinus ostia in children was safe and a significant number showed improvement of their SN-5 at 1 year of follow-up [9]. In a 2008 multicenter registry of balloon catheter sinusotomy outcomes for 1,036 patients, a total of 3,276 sinuses (an average of 3.2 sinuses per patient) underwent balloon catheter dilation. The average surgery time was 73 min. Of the 1,036 patients treated, 25 patients (2.4%) required revision of sinuses [10].

There have been very few published articles on the feasibility of sinus balloon dilation in the treatment of acute sinusitis and only a few case reports. The sinus balloon dilation treatment of acute sinusitis in children has not been reported in detail. Therefore, it has not been clearly defined whether acute sinusitis is suitable for surgery and what kind of operation should be chosen. For this case, we used the sinus balloon dilation to manage acute sphenoid sinusitis in the girl. The decision to operate was based on the following four factors, acute sphenoid sinusitis with impaired vision, short duration, no improvement, and the age of the child. In this procedure, the operating time for dilation of sphenoid sinus was 5 minutes. The overall operation time did not extend beyond 20 minutes. Moreover, no haemorrhage and pain happened intraoperatively or postoperatively. The patient's left vision was good on the first postoperative day. These reports and our case report have shown that balloon catheter dilation surgery was safe, rapid, and effective in selected patients with rhinosinusitis. In addition, some studies anecdotally reported that sinus balloon dilation effectively relieves sinus ostial obstruction with less postoperative pain, scarring, and bleeding than typically seen with traditional instruments [5]. Furthermore, balloon catheter dilation has tangible benefits such as a reduction in invasiveness of the intervention, hospital stay, recovery time, postoperative debridements, postoperative medications, and office follow-up visits [10, 11]. Provided that indications for sinus balloon dilation are selected correctly, with rich knowledge of the anatomy and appropriate surgical technique, this procedure can be achieved safely and rapidly.

## 4. Conclusions

Selected correctly, opportunities and ways for surgery are critical to patients. Balloon catheter dilation is a good choice for acute sphenoid sinusitis with severe orbital complication, especially in children. I think that this novel technique should promote discussion and be the preferred management in this condition.
